# Supplementary material for: Protecting larval fish at water intakes: hydraulic and biological evidence for the effectiveness of modern fish-protection screens
Source: Biol Open. 2025 Dec 17;14(12):bio062262. doi: 10.1242/bio.062262 (PMC12755069; doi:10.1242/bio.062262)
Supplement: Supplementary information [file biolopen-14-062262-s1.pdf]

Supplementary Eqn S1: Slot velocity calculation

Slot velocity (Sv) refers to the velocity of water as it passes through the open slots of a wedge-wire fish screen. Many international studies and guidelines use slot velocity instead of approach velocity. Therefore, to assist with comparison between our study and others and international guidelines, we estimated derived slot velocity from our direct measurements of approach velocity in the following way. The slot velocity is greater than the approach velocity due to the reduced area available for flow.

Slot velocity is calculated using the following equation.

Eqn S1: $$Sv = AV \times \left(\frac{S+B}{S}\right)$$

Where:
Sv : Slot velocity (m s^{-1})
AV : Approach velocity (m s^{-1})
S : Slot width (mm)
B : Bar width (mm)

Example Calculations

Two example calculations (based off our mean AV measurements are presented below using a slot width (S) of 2 mm and bar width (B) of 1.8 mm.

Case 1: AV = 0.089 m s^{-1}

$$Sv = 0.089 \times \left(\frac{2 + 1.8}{2}\right) = 0.1691$$

Case 2: AV = 0.203 m s^{-1}

$$Sv = 0.023 \times \left(\frac{2 + 1.8}{2}\right) = 0.3857$$
